# Promising PTFE-coating technology of Optimus-CVS™ stents: The new player for congenital heart disease interventions^☆^

**DOI:** 10.1016/j.ijcchd.2022.100323

**Published:** 2022-01-07

**Authors:** Raymond N. Haddad, Damien Bonnet, Jean-Marc Alsac, Sophie Malekzadeh-Milani

**Affiliations:** a M3C-Necker, Hôpital Universitaire Necker-Enfants Malades, AP-HP, Paris, France; b Department of Vascular Surgery, Hôpital Européen Georges-Pompidou, AP-HP, Paris, France; c Université de Paris, Paris, France

**Keywords:** Congenital heart disease, Stent implantation, Covered stent, Optimus, Innovation

## Abstract

**Background:**

The use of covered stents (CVS) in congenital heart disease (CHD) interventions is increasing but intrinsic properties of commercially available material remain not optimal. The Optimus-CVS™ (AndraTec GmbH, Koblenz, Germany) is a new balloon-expandable, non-premounted, Cobalt–Chromium stent with patented hybrid-cell design, innovative end-free Nano-PTFE sandwich-covering, and competitive deliverability features.

**Methods and results:**

Three patients with CHD received Optimus-CVS during December 2020 at our institution. Indications for CVS implantation were relief of complex aortic recoarctation (57 ​mm long Optimus-CVS-XL), treatment of intra-stent intimal proliferation in Fontan circulation (48 ​mm long Optimus-CVS-XXL), and as a bail-out for conduit rupture during pulmonary valve implantation (33 ​mm long Optimus-CVS-XXL). Procedures were successful and straightforward. Target diameter was achieved and stent shortening was minimal and as expected. No stent failure was recorded on short-term follow-up.

**Conclusions:**

Optimus-CVS appears to be a reliable high-performing CVS alternative in major CHD situations where CVS are needed. Optimus-CVS offers interesting construction characteristics, smart covering design, and the largest portfolio in terms of diameters and length optimizing lesion care and improving outcomes.

## Highlights

• The use of covered stents in CHD interventions is increasing and material development is needed.
• Optimus-CVS is a new covered stent alternative with competitive intrinsic properties.
• Stent efficacy and successful outcomes were proven in our preliminary experience.
• The smart covering design maximizes performance and confidence during manipulations.
• The largest portfolio in terms of diameters and lengths optimizes lesion care.

## 1 Introduction

Advances in the development of highly performing materials are required to match up the evolving widespread practice of covered stents (CVS) implantation in riskier anatomies, to expand the indications of transcatheter interventions, and to compensate for the technical failures of commonly available CVS [[1], [2], [3]]. The recently introduced Optimus-CVS™ (AndraTec GmbH, Koblenz, Germany) is a balloon-expandable, non-premounted, Cobalt–Chromium (CoCr) stent with patented hybrid-cell design, innovative technology of end-free Nano-PTFE sandwich-covering, and competitive deliverability features. This study aims to present the recent use of Optimus-CVS in the 3 major congenital heart disease (CHD) indications and define the impact of the new player in our field.

## 2 Methods

Consecutive patients with CHD receiving transcatheter implantation of Optimus-CVS at our institution during December 2020 were included in this study. Informed consent was obtained from each patient to perform the procedure and to use their clinical records. The study protocol conforms to the ethical guidelines of the 1975 Declaration of Helsinki as reflected in a priori approval by the institution's human research committee. Patients' clinical characteristics and procedural data were collected from the medical records. Standard safety, immediate, and short-term outcomes were assessed.

## 3 Results

### 3.1 Case no.1 (Fig. 1A)

A 20-year-old female patient (53 Kg/170 ​cm) with surgically repaired tetralogy of Fallot was referred for a stenotic dysfunctional 20 ​mm large Contegra tube. Calcified tube pre-stenting was performed across a 26-Fr femoral vein access. A 33 ​mm long Optimus-XXL bare-metal stent was deployed with a 24 ​mm large balloon-in-balloon (BIB)® (NuMED, Inc., Hopkinton, NY, USA). Contained rupture of the proximal conduit was immediately noticed on control angiography and controlled with another 33 ​mm long Optimus-CVS-XXL. An Edwards SAPIEN™ XT-23 valve (Edwards Lifesciences, Irvine, CA, USA) was successfully implanted during the same procedure with excellent 3 months outcomes.

### 3.2 Case no.2 (Fig. 1B)

A 31-year-old male patient (108 Kg/189 ​cm) was referred for recurrent aortic coarctation 18 years after surgical repair with a 14 ​mm large tube. He presented with therapy-resitant arterial hypertension and brachial-femoral pulse delay. Doppler ultrasound assessment confirmed the re-coarctation. Complementary imaging showed a 29 ​mm large ascending aorta narrowing down to 3.2 ​cm long/11 ​mm large tortuous coarctation, 15 ​mm distal to the origin of the neck vessels. A 57 ​mm long Optimus-CVS-XL was deployed across a 12-Fr femoral artery using a (20 ​mm x 4cm) AltoSa-XL® balloon (AndraTec GmbH Koblenz, Germany) inflated at 6 ATM. A 20 mm large high-pressure balloon achieved proximal and distal sections flaring. Final inner diameter at the smallest part increased from 10.5 to 15.7 ​mm. Stent shortening was 5.1% and the gradient dropped from 20 to 4 ​mmHg. Discharge and 3 months follow-up with TTE showed excellent outcomes. The patient is asymptomatic and the antihypertensive therapy was stopped.

### 3.3 Case no.3 (Fig. 1C)

A 33-year-old female patient (82 Kg/170 ​cm) with a palliated PA/IVS received a Melody valve in her non-fenestrated Fontan circulation in 2012 for severe lower limb venous insufficiency and had to be re-stented with an 8-Zig bare-metal CP-stent after 2 years for clinical deterioration. The patient was referred for intra-stent stenosis that was noted on control imaging. Pre-dilatation using a 22 ​mm large high-pressure balloon demonstrated significant recoil. A 48 ​mm long Optimus-CVS-XXL was implanted across a 12Fr jugular vein, using a 26 ​mm large BIB to exclude intra-stent intimal proliferation. The stent foreshortening was 6.4% and the lesion inner diameter increased from 17 to 25 mm. Post-dilatation was not required. Clinical outcomes were satisfactory.

## 4 Discussion

Indications for CVS implantation in the treatment of CHD are not well-defined but routine availability of this material has led to an expansion of stent implantation in more challenging high-risk anatomies [1]. However, there are concerns about the performance of commercially available CVS [[2], [3], [4]]. The CP stent® (NuMED Inc., Hopkinton, NY, USA) is the most commonly used CVS in our field. The poor quality of the covering implies precautious use during manipulations. Excessive stent shortening and PTFE-coating over-stretching have been seen with fully expanded CP stents in very dilated vessels negating many benefits of the CVS nature. Other CVS coming from the interventional radiology field have been recently introduced to the field. The response of the community has been varied and their use in CHD remains limited. Advanta™ V12 large-diameter stent (Getinge AB, Gothenburg, Sweden) is a stainless-steel CVS pre-mounted on non-compliant balloons that expands from 12 to 16 mm with 9 to 11-Fr compatibility. Interventionists were reluctant to its use because of secondary collapses. The use of LIFESTREAM® stent (Bard Peripheral Vascular, Inc., Tempe, AZ, USA) was limited to Potts creation. Both LIFESTREAM and Advanta share the same sandwich covering technology that was incriminated behind Advanta's poor radial force. The BeGRAFT aortic-stent-graft (Bentley InnoMed GmbH, Hechingen, Germany) is an open-cell CoCr platform, premounted on a proprietary single balloon, and designed with 9–14Fr compatibility and a wide range of diameters (12–24 ​mm, up to 30mm) and lengths (19–59 ​mm). It has recently been CE-marked but its clinical use has not been reported extensively. The BeGRAFT shares with CP stent the similar external covering design but the ePTFE layer is thinner (200 ​μm) and clamped by double stent struts at the ends. Its intrinsic properties are comparable to those of the CP stent but exceed those of its competitors in terms of radial force, foreshortening, and recoil.

The Optimus-CVS is a unique balloon-expandable, non-premounted stent that combines the advantages of all its competitors with novel advanced technologies to treat medium to extra-large lumens with extra safety. The superior HD CoCr MP35N alloy frame promises high radial strength and resistance to fracture required in the treatment of calcified conduits and severe re-coarctations. AndraTec's patented hybrid-cell design with omega-flex-connectors maximizes the flexibility and anatomical conformability of the stent required for better patient outcomes. It is noteworthy that AndraStent® (AndraMed, GmbH, Reutlingen, Germany) used to have a similar design. However, AndraStents only exist in bare-metal versions and a recent significant change occurred in their design from the flex segment to the straight-bridge design and the round edges towards a sharp squarer version. The metal struts of Optimus-CVS are sandwiched with inner and outer layers of Nano-ePTFE layers (Fig. 2). This highly performing covering design allows secured PTFE-attachment in challenging anatomies and during urgent manipulations in case of iatrogenic vascular leakage. It also offers a predictable behavior in response to intentional perforation to reanimate neck vessels [4]. The PTFE-coating is designed with unique end-free technology. One-half of a row of cells remains bare segments at both extremities of the stent. This new design combined with thermal bonding technology ensures optimal performance with the largest functional expansion range (12–28 ​mm, up to 32mm) and the lowest benchside foreshortening characteristics. It reduces the risk of PTFE-cover over hanging and/or damaging during expansion and also allows the stent to securely anchor the vessel walls, with limited recoil. Most importantly, this stent can be re-expanded in growing patients during follow-up to catch up adult vessel diameter. The lower profile of Optimus-CVS compared to other available CVS is a major advantage in children, particularly in arterial interventions. AndraTec recommends AltoSa-XL and AltoSa-XL-Gemini balloons for Optimus-CVS implantations, to profit from the lowest profile and smallest sheath compatibility on the market, but their performance in tortuous vessels remains not optimal. Finally, the large portfolio in terms of diameters and length of this non-premounted stent is valuable in small-volume centers as it allows to independently stock fewer stents in the armamentarium and freely use available delivery balloons maximizing the treatment of various anatomies.

**Fig. 1:**
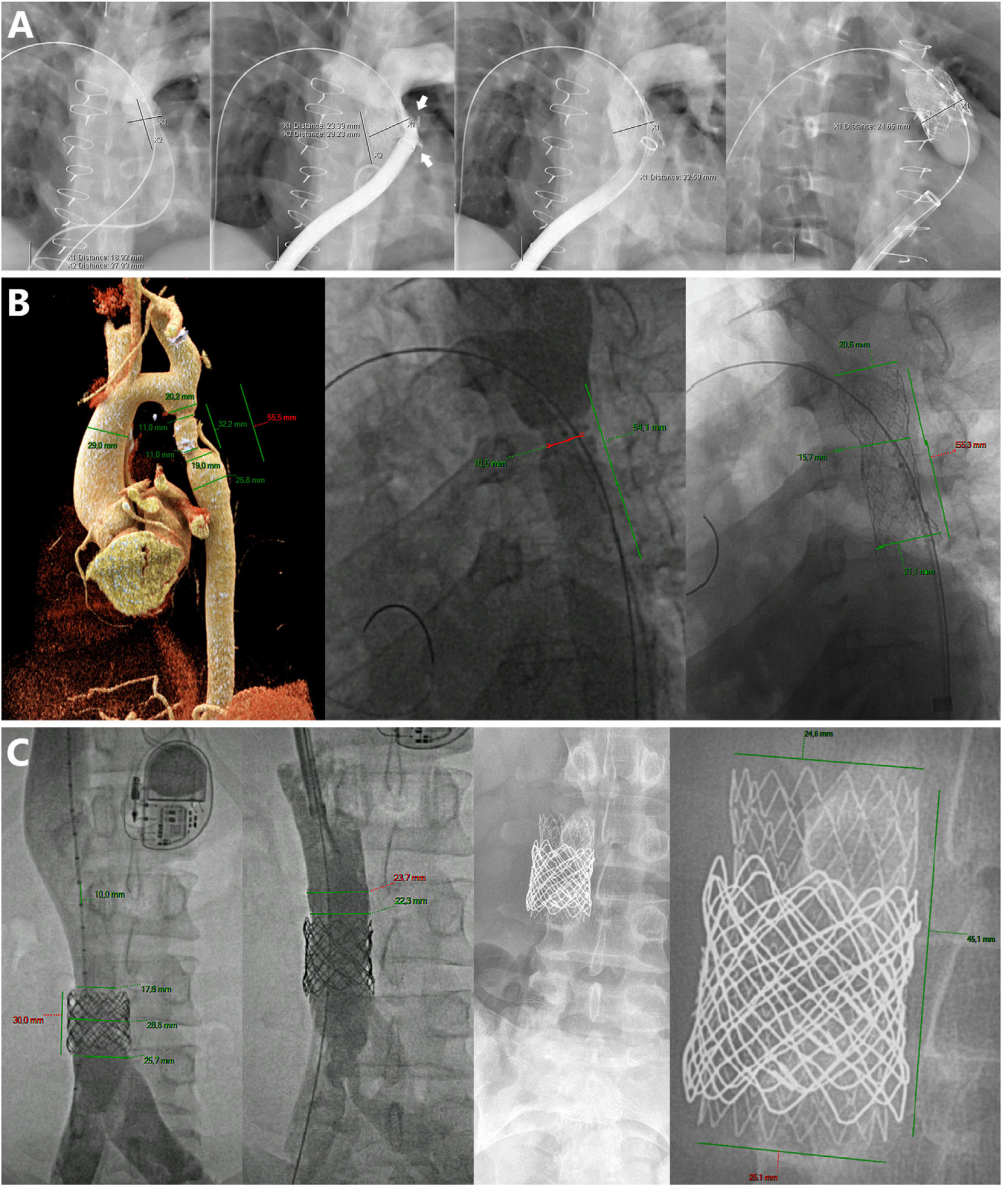
Detailed presentation of successful implantations of Optimus-CVS® stents in 3 different anatomies. (A) case 1, (B) case 2, (C) case 3.

**Fig. 2:**
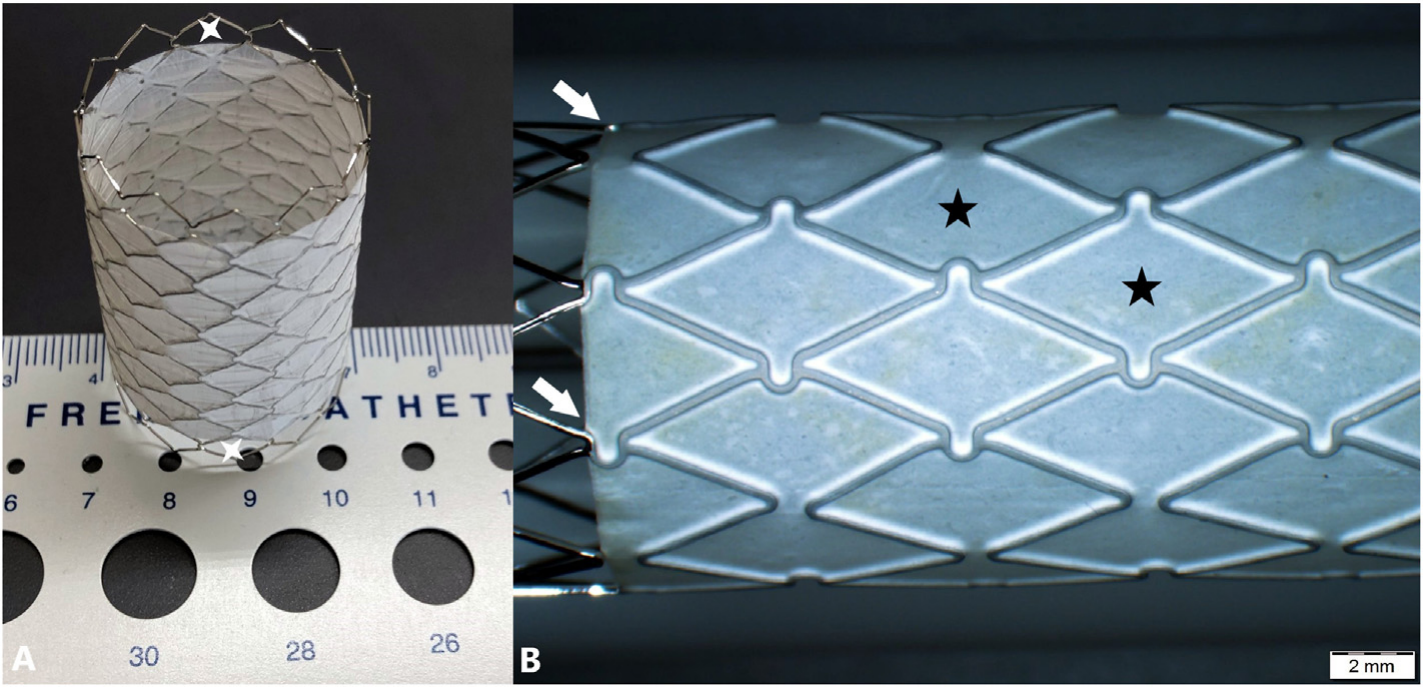
Optimus-CVS® stent. (A) End-free design with atraumatic round edges (white stars). (B) Thermal bonding of surface sealing (white arrows) gives the impression that the PTFE coating has been pressed onto the metal struts. Note the stent's special hybrid design with omega-flex-connectors (black stars).

### 4.1 Limitations

Balloon-expandable CVS have enough radial strength to treat stenotic lesions and offer protection from aneurysm formation in the covered vessel segments. However, aneurysms can still occur at the extremities of the stents, especially if the balloon diameter is larger than adjacent vessel walls, and self-expanding CVS may be better for the treatment of post-surgical aneurysms [5]. Compared to self-expanding CVS in the treatment of vessel rupture, the recoil inherent to balloon-expandable CVS is a serious disadvantage that should be always taken into consideration in this setting [6].

Covered stenting for sinus venosus atrial septal defects closure is a newly developed intervention that is mainly performed using 10-zig covered CP stents of lengths between 5 and 8 ​cm [7]. The promising characteristics of the Optimus-CVS make this new stent suitable for this procedure. To date, the maximal available length (i.e. 57 ​mm) seems short for this indication and longer lengths are expected before its use in this indication.

## 5 Conclusion

Our preliminary experience shows that Optimus-CVS has the potential to provide a very competitive and reliable medium to extra-large bore CVS alternative in major CHD situations where CVS are needed. Its innovative design and construction characteristics are very encouraging and the implantation procedure is safe, straightforward, and efficacious. A multicentre registry is required to assess this new technology.

## Funding

None.

## Authors’ contributions

RH collected all clinical and imaging data, designed illustrative material, and took the lead in writing the entire manuscript. All authors discussed the results, read, and approved the final version of the manuscript.

## Declaration of competing interest

The authors declare that they have no known competing financial interests or personal relationships that could have appeared to influence the work reported in this paper.
